# The fairness of human resource management practices: an assessment by the justice sensitive

**DOI:** 10.3389/fpsyg.2024.1355378

**Published:** 2024-03-26

**Authors:** Victor Y. Haines, David Patient, Sylvie Guerrero

**Affiliations:** ^1^School of Industrial Relations, University of Montreal, Montreal, QC, Canada; ^2^Vlerick Business School, Ghent, East Flanders, Belgium; ^3^École des sciences de la gestion (ESG), Université du Québec à Montréal (UQAM), Montreal, Montreal, QC, Canada

**Keywords:** fairness, human resource management, pay-for-performance, justice sensitivity, equity principle, systemic justice

## Abstract

**Introduction:**

Although fairness is a pervasive and ongoing concern in organizations, the fairness of human resource management practices is often overlooked. This study examines how individual differences in justice sensitivity influence the extent to which human resource management practices are perceived to convey principles of organizational justice.

**Methods:**

Analysis was performed on a matching sample of 283 university students from three academic units in two countries having responded at two time points. Justice sensitivity was measured with the 40-item inventory developed and validated by Schmitt et al. (2010). Respondents were instructed to indicate to what extent each of 61 human resource management practices generally conveys principles of organizational justice.

**Results:**

Justice sensitivity was positively associated with subsequent assessments of the justice contents of human resource management practices. The distinction between self-oriented and other-oriented justice sensitivities was helpful in determining perceptions of these human resource management practices and of a subset of pay-for-performance practices.

**Discussion:**

The findings inform current research about the meanings borne by human resource management practices, and also increase understanding of entity judgment formation as an important aspect of systemic justice.

## Introduction

Consequences of employee positive fairness perceptions include higher job satisfaction, organizational commitment, and performance (Cohen-Charash and Spector, 2001). This provides a compelling argument in favor of human resource management practices that are clearly justice-oriented, such as employee participation and dispute resolution mechanisms. However, because other human resource management practices, such as selective hiring or skills development, are less clearly justice-oriented, it can be challenging to determine which practices to implement to foster positive fairness perceptions. Additionally, individuals with different sensitivities to injustice may respond differently to specific human resource management practices. Some employees experience strong negative emotions when exposed to unfair practices, whereas others are more indifferent (Lovaš and Wolt, 2002). Some care especially about (in)justice affecting themselves, whereas others mostly want others to be treated fairly (Schmitt et al., 2005, 2010).

The aim of this study is to determine how individual differences in justice sensitivity influence the extent to which human resource management practices are perceived to convey principles of organizational justice. We investigate the perceived justice of a wide range of human resource management practices, with a particular focus on measures relating to pay-for-performance. Our reason for focusing on the reward system is its clear fairness implications in terms of the principles that guide the allocation of resources whereby individuals and groups in organizations feel that they receive what they deserve.

This research can increase our understanding of why and how human resource practices are interpreted as fair, and why individuals with self-oriented versus other-oriented justice sensitivity vary in their assessments of them. We thereby extend research about the meanings borne by human resource management practices (*cf.* Bowen and Ostroff, 2004; Nishii et al., 2008). However, rather than focus on how the message is conveyed (e.g., signal strength), we examine how an important justice-related disposition – justice sensitivity – can influence how these management practices are perceived. This study thereby builds on the idea that the way an object is perceived depends in part on the characteristics of the receiver (Brunswik, 1956), and responds to calls to consider the influence of individual differences on perceptions of human resource management practices (Nishii et al., 2008; Wang et al., 2019). However, rather than investigate perceptions of the availability of human resource management practices (e.g., Den Hartog et al., 2013; Jiang et al., 2017), this study asks a more challenging question about the perceived justice contents of these practices.

Much of the justice sensitivity research has addressed questions of social psychology in laboratory situations with experimental designs involving (in)justice (e.g., Fetchenhauer and Huang, 2004; Lotz et al., 2011). The current investigation extends justice sensitivity research about social interactions and moral dilemmas to include justice-oriented social structures (i.e., management practices). In doing so, this study contributes to justice research more broadly regarding what is considered fair or the phenomenology of justice (Mikula et al., 1990). In addition, rather than focus on events occurring within close personal relationships of equal power, this study investigates the perceived fairness of stable social cues that promote justice and that exist in task-oriented relationships of unequal power (i.e., human resource management practices). This structural perspective is expected to provide a clearer view of what might be considered components of systemic justice that define organizations in terms of their justice orientation (Sheppard et al., 1992; Beugré and Baron, 2001).

This study may also guide strategic human resource management research by showing that human resource management practices not only have meanings that relate to ability, motivation, and opportunity (Combs et al., 2006; Lepak et al., 2006; Fu et al., 2017), but also to a broader system-level concern for organizational justice (Haines III et al., 2018). In mapping the social organizational context in which justice events unfold (*cf.* Keeley, 1988; Hollensbe et al., 2008; Rupp and Paddock, 2010; Cooper and Scandura, 2015), the current study complements work by Schminke et al. (2000, 2002) that investigated the influence of organizational structure (e.g., centralization, formalization) on entity fairness perceptions. This trajectory should not only clarify the deeper meanings attributed to human resource management practices (i.e., components of the social organizational context) – and, in particular, pay-for-performance practices – but also help establish new connections with employer branding research, wherein organizations are characterized by their key values and guiding principles (Dell and Ainspan, 2001; Edwards, 2010). To the extent that key values and guiding principles are transmitted by human resource management practices, then it becomes most relevant to investigate them from a justice perspective.

This study also considers the effects of preference for the equity principle on perceptions of human resource management practices. By considering effects of both justice sensitivity and preference for the equity principle, we improve understanding of how employees assess the fairness of human resource management practices, including pay-for-performance practices (e.g., St-Onge, 2000; Chang and Hahn, 2006).

## Justice sensitivity

Social justice research has documented stable individual differences in justice sensitivity (Schmitt et al., 1995, 2010; Baumert and Schmitt, 2009), a social-cognitive personality trait that reflects an individual’s concern for justice. Four facets of justice sensitivity were identified: victim, observer, beneficiary, and perpetrator sensitivities. Initial research focused on the perspective of victims (Schmitt et al., 1995; Schmitt and Mohiyeddini, 1996; Mohiyeddini and Schmitt, 1997; Schmitt and Dörfel, 1999), but subsequent conceptual refinements have drawn on perspective effects in justice judgments (Mikula, 1994) to develop additional scales for perpetrators and observers (Schmitt et al., 2005) as well as beneficiaries (Schmitt et al., 2010). The anger experienced by victims was replaced by guilt in the perpetrator scale and by moral outrage in the observer scale. These four facets of justice sensitivity have been investigated in terms of frequency of perceived injustice, intensity of emotional reactions to injustice, intrusiveness of thoughts about perceived injsutice, and motivation to restore justice.

With regard to the moral emotions experienced in the face of injustice, the four facets differ in important ways. Victims experience anger, while observers experience moral outrage, and beneficiaries and perpetrators experience guilt. Although all four facets capture individual differences, victim sensitivity reflects a self-oriented concern with being personally treated unfairly by others. In contrast, observer, beneficiary, and perpetrator sensitivities all relate to a genuine concern for the justice experienced by others (Fetchenhauer and Huang, 2004; Schmitt et al., 2010; Lotz et al., 2011; Decety and Yoder, 2016; Bondü et al., 2022). The victim-sensitive are motivated by egoistic concerns whereas observer, beneficiary, and perpetrator facets share altruistic concerns.^1^

At the core of justice sensitivity are perceptual and motivational processes (Schmitt et al., 2005). Baumert et al. (2011) found that the cognitive (perspective taking) and motivational (empathetic concern) components of empathy predicted overall justice sensitivity whereas the emotional (affective sharing) component did not. Consistent with the social-cognitive view whereby each personality trait has its own information processing patterns (Rusting, 1998), overall justice sensitivity is expected to guide information processing relating to justice-oriented social cues. As stated by Baumert and Schmitt (2009), “the accessibility of injustice concepts seems to be the more fundamental cognitive mechanism in JS [justice sensitivity]” (p. 10). Highly justice sensitive individuals are hyper-vigilant to justice-related cues (Baumert et al., 2011; Bondü and Inerle, 2020) and tend to anticipate unjust situations (Fetchenhauer and Huang, 2004; Bondü, 2018). Injustice is more salient to the justice sensitive, and this increases excitation and activation of this construct in interpreting situations (Higgins and Brendl, 1995). Therefore, as proposed in research on personality and cognition (e.g., Cantor, 1981), individual variability in justice sensitivity is associated with construct accessibility.

### Justice sensitivity and human resource management

How individual interpretations of complex social structures are shaped by justice sensitivity remains an underdeveloped area. Individuals more concerned with justice, the justice sensitive, are generally expected to perceive a greater justice orientation than the less justice sensitive in human resource management practices that distribute socially valued resources (e.g., employment, income, training and development) by establishing clear and consistent rules and procedures (Kroon et al., 2009). Moreover, by way of the adoption of clear rules and procedures, human resource management practices are at times conceived as participating in a process of formalization (e.g., Kotey and Slade, 2005; López et al., 2019). The adoption of human resource management practices should therefore, as a general rule, be associated with more procedural clarity and fairness. Even if there is some room for malevolence in the application of human resource management practices (Longenecker et al., 1987; Nishii et al., 2008; De Clercq et al., 2019), they are generally developed and implemented with a concern for justice (Folger and Cropanzano, 1998) and with an inherent fairness motive (Koys, 1991). This orientation is conveyed in the curriculum and professional standards that guide the practice of human resource management.^2^ The concern for justice is also conveyed in several high-performance and commitment-oriented human resource management practices geared at promoting employee involvement and voice (Colvin, 2006). In this light, it might come as no surprise that human resource management practices are often conceptualized as resources (e.g., Peters et al., 2014).

Because the personal importance of justice is greater for the justice sensitive, their perceptual and motivational processes should result in them viewing human resource management practices as more justice-oriented. They are, as such, high in justice sensitivity and thereby predisposed to notice the justice contents of human resource management practices.

*Hypothesis 1*: There will be a positive association between overall justice sensitivity and the extent to which human resource management practices are perceived to convey principles of organizational justice.

Alternatively, because the justice sensitive have a lower threshold for perceiving breaches of justice, they may be more inclined to perceive some human resource management practices as not or not sufficiently reflecting justice concerns. We will consider this possibility further on as we address the particulars of pay-for-performance.

### Self and other sensitivities and human resource management

In line with developments in social justice research (e.g., Schlösser et al., 2018; Strauß and Bondü, 2022), assessments of the justice orientation of human resource management practices are expected to be influenced by self- versus other-oriented justice sensitivities. Whereas victim sensitivity represents self-oriented justice sensitivity, the other-oriented sensitivities are often studied together (e.g., Fetchenhauer and Huang, 2004; Schlösser et al., 2018; Strauß and Bondü, 2022), or with only one of three facets used to represent an other-orientation (e.g., Tham et al., 2019). Using the first approach, we combined observer, beneficiary, and perpetrator sensitivities to represent the other-orientation. The analysis will therefore relate justice for self (i.e., victim sensitivity) and justice for others (i.e., observer, beneficiary, and perpetrator sensitivities combined) to the extent to which human resource management practices are perceived to convey principles of organizational justice.

The victim sensitive care about justice when they feel they have been unfairly disadvantaged. Their concern for justice is self-oriented and therefore focused on advantages or disadvantages to themselves (Schmitt et al., 1995). They have a suspicious mindset and are motivated to avoid being exploited (Gollwitzer et al., 2005). The victim sensitive also tend to expect unjust outcomes in ambiguous situations (Maltese et al., 2016). The egoistic motivation of the self-sensitive may therefore result in them perceiving as less justice-oriented human resource management practices that establish clear, consistent rules and procedures for the collective.

In contrast, other-oriented sensitivities are likely to see a justice orientation in a wider range of human resource management practices. Other-oriented justice sensitivities share a genuine concern for the just treatment of others (Fetchenhauer and Huang, 2004; Schmitt et al., 2010; Lotz et al., 2011; Decety and Yoder, 2016), manifested in prosocial dispositions (Schmitt et al., 2005) and behavior (Fetchenhauer and Huang, 2004; Gollwitzer et al., 2005, 2009). Other-oriented justice sensitivities are therefore expected to view as more just human resource management practices that seek to establish clear and consistent rules and procedures for the collective.

*Hypothesis 2*: There will be a stronger positive association between other-oriented justice sensitivity (relative to self-oriented justice sensitivity) and the extent to which human resource management practices are perceived to convey principles of organizational justice.

#### Justice sensitivities and pay-for-performance

By their very nature, organizational pay-for-performance practices involve resource distributions according to this principle of equity. Components of the reward system can be individualistic or collectivistic, with the former (e.g., merit-based pay) allocating benefits according to individual effort and the latter (e.g., team rewards) allocating benefits according to collective effort.

There are several reasons why a positive relationship between overall justice sensitivity and positive perceptions regarding justice of pay-for-performance can be expected. First, overall justice sensitivity has a collectivistic (versus individualistic) orientation due to that fact that three of its four facets focus on the experiences of others, rather than of the self. Because there are collectivistic components in both justice sensitivity (namely in the observer, beneficiary, and perpetrator sensitivities) and in pay-for-performance practices (e.g., team rewards), we expect overall justice sensitivity to relate positively to assessments regarding the justice orientation of pay-for-performance practices. Second, as with other human resource management practices, pay-for-performance practices seek to establish clear and consistent procedures. This procedural aspect of pay-for-performance practices also supports a positive association between overall justice sensitivity and perceptions of the justice of pay-for-performance practices.

*Hypothesis 3*: There will be a positive association between overall justice sensitivity and the extent to which pay-for-performance practices are perceived to convey principles of organizational justice.

The individualistic and collectivistic orientations of pay-for-performance practices might best be disentangled by also looking at self- and other-oriented sensitivies. To the extent that pay-for-performance involves the allocation of benefits according to effort, the individual stands to benefit regardless of whether or not the effort under scrutiny is that of the individual or of the collective. The principle of equity is self-focused (Sabbagh et al., 1994) and therefore so are the pay-for-performance practices that apply this principle. The concern for justice of the victim sensitive is self-oriented and therefore focused on their own advantages or disadvantages (Schmitt et al., 1995). The egoistic motivation of the self-sensitive may therefore have them perceive more justice in pay-for-performance practices because they stand to benefit personally from the availability of such practices.

*Hypothesis 4*: There will be a stronger positive association between self-oriented justice sensitivity (relative to other-oriented justice sensitivity) and the extent to which pay-for-performance practices are perceived to convey principles of organizational justice.

The benefits/effort ratio is a defining characteric of pay-for-performance practices. Some practices, however, apply this ratio to the individual and others to the collective. Examining individualistic (e.g., merit-based pay) and collectivistic (e.g., team rewards) pay-for-performance practices seperately opens a pathway to better understanding how justice sensitivities relate to the perceived justice of differential applications of the merit principle. Henceforth, self-oriented justice sensitivity was expected to be more strongly associated with positive assessments of the justice of individualistic pay-for-performance practices and other-oriented justice sensitivity was expected to be more strongly associated with positive assessments of the justice contents of collectivistic pay-for-performance practices.

*Hypothesis 5*: There will be a stronger positive association between self-oriented justice sensitivity (relative to other-oriented justice sensitivity) and the extent to which individualistic pay-for-performance practices are perceived to convey principles of organizational justice.

*Hypothesis 6*: There will be a stronger positive association between other-oriented justice sensitivity (relative to self-oriented justice sensitivity) and the extent to which collectivistic pay-for-performance practices are perceived to convey principles of organizational justice.

#### Justice sensitivities and preference for the equity principle

Deutsch (1975) defined the principle of equity^3^ as the allocation of benefits according to effort. A personal preference for allocating outcomes and rewards to those with the highest inputs, often in proportion to inputs, is deemed a preference for the equity principle (*cf.* Davey et al., 1999). In terms of distributive rules (Deutsch, 1975), a preference for the equity principle conveys a preference for the equity rule relative to equality and need rules.

We included preference for the equity principle in hypothesis testing with the aim of testing the incremental validity of justice-oriented social structures (i.e., human resource management practices). In investigating the distinctive effect of justice sensitivity on the justice orientation of human resource management practices, it was deemed important to show the effects of justice sensitivity over and above preference for the equity principle. Both justice sensitivity and preference for the equity principle have a clear justice orientation and both constructs reflect individual differences. Considering that justice sensitivity presents a relatively recent conceptualization capturing individual differences with a clear justice orientation, we deemed it important to determine its relative contribution to statistical variance in assessments of human resource management practices. We therefore propose to examine the incremental contribution of justice sensitivity, over and above equity principle adherence with regard to the perceived justice-orientation of human resource management practices. A first regression equation will test the hypothesized associations without preference for the equity principle and a second equation will test them with this variable and two control variables included.

With regards to the two fundamental facets of justice sensitivity, one might also rightly question the incremental validity of self-oriented justice sensitivity relative to preference for the equity principle. Both of these individual differences are self-focused (Sabbagh et al., 1994; Schmitt et al., 2005), so that the victim sensitive and individuals with a stronger preference for the equity principle should see more justice in the allocation of benefits according to individual effort. The question of the incremental validity of self-oriented justice sensitivity beyond the variance captured by preference for the merit principle with regards to assessments of the justice contents of individualistic pay-for-performance practices will therefore be scrutinized. By including preference for the equity principle in the multivariate equations, the analysis will, as such, test the incremental validity of justice sensitivity and its fundamental perspectives (i.e., self- versus other-oriented sensitivities) with regards to assessments of the justice contents of human resource management practices and subsets of individualistic and collectivistic pay-for-performance practices.

## Methods

### Data

Students from two business schools and from a department of industrial relations were invited to participate in this study during their regular academic semester. In advance of the classes in which questionnaires were completed, the agreement of the instructor was obtained. The same procedure was followed in the three classes (one from each institution).

Data were collected at two time intervals so as to limit response fatigue and to lessen concerns about common method variance. At Time 1, a research assistant explained the study and distributed questionnaires and consent forms. Participants completed the survey and consent form, put both in an envelope, and then wrote their name on and submitted the envelope. The same procedure was implemented at Time 2, 1 week later. A matching number was assigned to each participant and the envelopes with participant names subsequently discarded, to maintain confidentiality.

A total of 490 students participated in the study at Time 1 and 419 at Time 2. All students present at Time 2 were invited to complete the questionnaire regardless of whether they had participated at Time 1. Although students were informed by the research assistant that they had the option of handing in a blank survey within a sealed envelope to avoid being singled out, almost all students present in class completed the questionnaire. Because of variations in the students that were present in class at Time 1 or Time 2, the analysis proceeded with 283 matching responses with complete data on the variables of interest. Of these, 67% were women and 64% were enrolled in a business school. Almost all respondents (96%) had some work experience with an average tenure of 5.13 years in paid employment. At the time of the survey, 66% of respondents reported being currently employed.

### Measures

A French version of the justice sensitivity inventory (Faccenda et al., 2008), was extended to include perpetrator sensitivity. The other scales used in this study were translated to French by a first professional translator and then back to English by a second professional translator (Brislin, 1970). Discrepancies between the original English version and the retranslated English version were discussed by two bilingual members of the research team, resulting in minor modifications to the French version.

Justice sensitivity, preference for the equity principle, gender, and academic unit were collected at Time 1. The perceived justice of human resource management practices was assessed at Time 2.

#### Justice of human resource management practices

In the process of developing a taxonomy of high-performance work practices, Posthuma et al. (2013) compiled a comprehensive list of 61 human resource management practices grouped into eight categories.^4^ From this list, we removed one practice because it directly implied justice in its application (i.e., equitable pay process). Another human resource management practice was removed because it addressed a broad managerial concern rather than a specific practice (i.e., turnover, retention, and exit management). Finally, two important practices were added to the list (i.e., health and safety measures, work-life balance flexibility).

Respondents were instructed to indicate to what extent each of the human resource management practices generally conveys principles of organizational justice, including distributive, procedural, interpersonal, and informational justice. It is important to note that given their area of study and past academic experience, study participants had all previously taken courses that addressed and defined organizational justice, as well as its distributive, procedural, interpersonal, and informational dimensions. The response format ranged from 1 (*not at all*) to 4 (*to a great extent*). Because the first hypothesis is based on the expectation that the justice sensitive will detect more justice in more human resource management practices, individual ratings of the 61 human resource management practices included in this modified index were averaged to obtain an overall score of the extent to which they are perceived as conveying principles of organizational justice.

#### Justice of pay-for-performance practices

The pay-for-performance index included the eight compensation practices that specifically included variable pay or pay-for-performance. These practices all included a benefit/effort ratio in one form or another (e.g., profit or gain sharing). Three of the eight pay-for-performance practices were used to assess the justice of individualistic pay-for-performance practices: “Formal appraisal for pay,” “pay-for-performance,” and “bonuses or cash for performance.” Three of the eight pay-for-performance practices were used to assess the justice of collectivistic pay-for-performance practices: “Profit or gain sharing,” “group-based pay,” and “employee stock ownership.”

#### Justice sensitivity

Justice sensitivity was measured with the 40-item inventory developed and validated by Schmitt et al. (2010). It includes 10 items for each of the four sensitivity perspectives. The following prompt preceded the four sensitivity perspectives: “How do you react in unfair situations? People react quite differently in unfair situations. How about you?” Then, for victim sensitivity: “First, we will look at situations to the advantage of others and to your own disadvantage.” This prompt was followed by the 10 items that assess victim sensitivity (e.g., “It bothers me when others receive something that ought to be mine”). Observer, beneficiary, and perpetrator sensitivities were measured with prompts and items adapted to each perspective. For instance, for observer sensitivity: “Now, we will look at situations in which you notice or learn that someone else is being treated unfairly, put at a disadvantage, or used” (e.g., “It bothers me when someone gets something they do not deserve”).

Responses were recorded on a scale ranging from 0 (*not at all*) to 4 (*exactly*). The reliability coefficient (alpha) for the 40-item inventory was 0.93. Self-oriented justice sensitivity was measured with the 10 items that take the victims perspective (alpha = 0.82). Other-oriented justice sensitivity was assessed with the 30 items that take the observer, beneficiary, and perpetrator perspectives (alpha = 0.94).

#### Preference for the equity principle

Respondents were asked to indicate their level of agreement with a single item**: “**When resources are allocated among people, the person with the highest inputs (e.g., effort, ability, qualifications) should get the highest outcomes.” Much like the preference for merit scale (Davey et al., 1999), this measure assumes that there are individual differences in preferences for distributive justice rules - which include equity, equality, and need (Deutsch, 1975) – and that some people adhere more to an equity rule. The responses were recorded on a scale ranging from 0 (*strongly disagree*) to 7 (*strongly agree*).

#### Control variables

Research, including examination of brain activation differences, have found stronger responses by females than males to procedural and distributive justice information (Dulebohn et al., 2016). We therefore controlled for gender as either man (0) or woman (1). Given the possibility that different demographics and belief systems may differentiate students enrolled in social sciences from those in business schools, the analysis also controlled for the type of academic department (industrial relations = 0, business school = 1).

## Results

Table 1 presents the descriptive statistics and correlations between the study variables. The regression equations reported in Table 2 include the justice sensitivity variables (Model 1). The second model includes preference for the equity principle, gender, and academic unit (Model 2). This follows the recommendation to test associations both with and without control variables (Aguinis and Vandenberg, 2014).

**Table 1:** Descriptive statistics and correlations between the study variables.

		*M*	*SD*	1	2	3	4	5	6	7	8	9
1.	Overall HRM ^a^	3.25	0.34									
2.	Pay-performance ^b^	3.04	0.42	0.58								
3.	Individualistic pay ^c^	3.22	0.51	0.39	0.75							
4.	Collectivistic pay ^d^	2.83	0.59	0.46	0.77	0.28						
5.	Overall JS	3.42	0.65	0.24	0.19	0.05	0.23					
6.	Self-oriented JS	3.30	0.80	0.18	0.21	0.17	0.15	0.61				
7.	Other-oriented JS	3.46	0.74	0.22	0.15	0.00	0.22	0.95	0.34			
8.	Preference equity ^e^	3.35	1.50	0.08	0.25	0.23	0.14	0.14	0.25	0.06		
9.	Gender ^f^	0.67	0.47	0.04	−0.09	−0.08	−0.05	0.28	0.17	0.26	−0.08	
10.	Academic unit ^g^	0.64	0.48	−0.11	−0.07	−0.14	−0.08	−0.25	−0.27	−0.19	0.04	−0.22

^a^Justice of all human resource management practices. ^b^Justice of pay-for-performance. ^c^Justice of individualistic pay-for-performance. ^d^Justice of collectivistic pay-for-performance. ^e^Preference for the equity principle. ^f^Man = 0, woman = 1. ^g^Industrial relations = 0, business school = 1. JS, justice sensitivity. All correlation coefficients with an absolute value of 0.14 or higher are significant at *p* < 0.05.

**Table 2:** Regression coefficients of justice of human resource management practices on justice sensitivities.

	Model 1				Model 2	
	*B*	*β*	*SE B*	*B*	*β*	*SE B*
Constant	2.81^***^		0.11	2.82^***^		0.13
Overall JS	0.13^***^	0.24^***^	0.03	0.12^***^	0.23^***^	0.03
Preference equity ^a^				0.01	0.05	0.01
Gender ^b^				−0.02	−0.03	0.04
Academic unit ^c^				−0.04	−0.06	0.04
*R^2^*	0.06^***^			0.07^***^		
*ΔR^2^*				0.01		
Constant	2.79^***^		0.11	2.81^***^		0.13
Self-oriented JS	0.05^*^	0.12^*^	0.03	0.04	0.10	0.03
Other-oriented JS	0.08^**^	0.18^**^	0.03	0.08^**^	0.18^**^	0.03
Preference equity ^a^				0.01	0.04	0.01
Gender ^b^				−0.03	−0.03	0.05
Academic unit ^c^				−0.04	−0.06	0.04
*R^2^*	0.06^***^			0.07^**^		
*ΔR^2^*				0.01		

^a^Preference for the equity principle. ^b^Man = 0, woman = 1. ^c^Industrial relations = 0, business school = 1. JS, justice sensitivity. ^*^p < 0.05. ^**^p < 0.01. ^***^p < 0.001.

The first hypothesis proposed a positive association between justice sensitivity and the perceived justice of human resource management practices. The regression coefficient indicates that higher overall justice sensitivity relates to more positive evaluations of justice-orientation for the broad set of human resource management practices. This association remains significant when preference for the equity principle, gender, and academic unit are included in the regression equation. Thus, the first hypothesis was supported.

The second hypothesis proposed a stronger positive association between other-oriented justice sensitivity and the perceived justice of human resource management practices. When preference for the equity principle, gender, and academic unit were included as covariates in the regression equation, self-oriented justice sensitivity (i.e., victim sensitivity) did not significantly relate to assessments of the justice-orientation of human resource management practices. Other-oriented justice sensitivity, however, remained positively and significantly associated with the perceived justice of human resource management practices. Thus, the second hypothesis was also supported.

Table 3 shows that both overall justice sensitivity and preference for the equity principle were positively related to the perceived justice-orientation of pay-for-performance practices. This provided support for the third hypothesis and showed the distinctive contribution of justice sensitivity over and above preference for the equity principle in predicting assessments of the justice-orientation of pay-for-performance practices. Interestingly, gender was significantly associated with the perceived justice-orientation of pay-for-performance practices. Women perceived less justice in pay-for-performance practices.

**Table 3:** Regression coefficients of justice of pay-for-performance practices on justice sensitivities.

	Model 1				Model 2	
	*B*	*β*	*SE B*	*B*	*β*	*SE B*
Constant	2.62^***^		0.13	2.41^***^		0.16
Overall JS	0.12^**^	0.19^**^	0.04	0.12^**^	0.18^**^	0.04
Preference equity ^a^				0.06^***^	0.23^***^	0.02
Gender ^b^				−0.11^*^	−0.13^*^	0.05
Academic unit ^c^				−0.05	−0.05	0.05
*R^2^*	0.04^**^			0.11^***^		
*ΔR^2^*				0.07^***^		
Constant	2.53^***^		0.13	2.38^***^		0.16
Self-oriented JS	0.09^**^	0.18^**^	0.03	0.07^*^	0.13^*^	0.03
Other-oriented JS	0.05	0.09	0.04	0.07^*^	0.13^*^	0.04
Preference equity ^a^				0.06^***^	0.21^***^	0.02
Gender ^b^				−0.12^*^	−0.13^*^	0.05
Academic unit ^c^				−0.04	−0.04	0.05
*R^2^*	0.05^***^			0.11^***^		
*ΔR^2^*				0.06^***^		

^a^ Preference for the equity principle. ^b^Man = 0, woman = 1. ^c^Industrial relations = 0, business school = 1. JS, justice sensitivity. ^*^p < 0.05. ^**^p < 0.01. ^***^p < 0.001.

The fourth hypothesis predicted a stronger positive association between self-oriented justice sensitivity relative to other-oriented justice sensitivity and assessments of the justice-orientation of pay-for-performance practices. This was partially supported (Table 3) by the stronger positive association between self-oriented justice sensitivity and the perceived justice-orientation of pay-for-performance practices when preference for the equity principle, gender, and academic unit were not included as covariates (Model 1). However, when these covariates were included, the predictive power of self-oriented justice sensitivity was reduced (Model 2), such that the fourth hypothesis was no longer supported.

Hypotheses 5 and 6 provided an opportunity to untangle the influences of preference for the equity principle and individualistic/collectivistic justice sensitivities on perceptions regarding the justice of pay-for-performance practices. Hypothesis 5 predicted a stronger positive association between self-oriented justice sensitivity (i.e., victim sensitivity) relative to other-oriented justice sensitivity and the perceived justice-orientation of individualistic pay-for-performance practices. As shown in Table 4, overall justice sensitivity was not significantly associated with assessments of the justice-orientation of individualistic pay-for-performance practices. The results, however, support the predicted stronger positive association between self-oriented justice sensitivity relative to other-oriented justice sensitivity and the perceived justice of individualistic pay-for-performance practices. However, the strength of this association was reduced and only marginally significant (*p* < 0.10) when preference for the equity principle, gender, and academic unit were added to the equation.

**Table 4:** Regression coefficients of justice of individualistic pay-for-performance practices on justice sensitivities.

	Model 1				Model 2	
	*B*	*β*	*SE B*	*B*	*β*	*SE B*
Constant	3.08^***^		0.16	2.97^***^		0.19
Overall JS	0.04	0.05	0.05	0.01	0.01	0.05
Preference equity ^a^				0.08^***^	0.23^***^	0.02
Gender ^b^				−0.11^†^	−0.10^†^	0.07
Academic unit ^c^				−0.17^**^	−0.16^**^	0.06
*R^2^*	0.00			0.08^***^		
*ΔR^2^*				0.08^***^		
Constant	2.97^***^		0.17	2.91^***^		0.20
Self-oriented JS	0.12^**^	0.19^**^	0.04	0.07^†^	0.11^†^	0.04
Other-oriented JS	−0.04	−0.06	0.04	−0.03	−0.05	0.04
Preference equity ^a^				0.07^***^	0.20^***^	0.02
Gender ^b^				−0.11^†^	−0.11^†^	0.07
Academic unit ^c^				−0.16^*^	−0.15^*^	0.07
*R^2^*	0.03^*^			0.09^***^		
*ΔR^2^*				0.06^***^		

^a^Preference for the equity principle. ^b^Man = 0, woman = 1. ^c^Industrial relations = 0, business school = 1. JS, justice sensitivity. ^*^p < 0.05. ^**^p < 0.01. ^***^p < 0.001. *^†^p* < 0.10.

Hypothesis 6 predicted a positive association between other-oriented justice sensitivity relative to self-oriented sensitivity and the perceived justice-orientation of collectivistic pay-for-performance practices. Table 5 shows strong support for Hypothesis 6 even after preference for the equity principle, gender, and academic unit were included in the analysis. Also reported is the non-significant association between self-oriented justice sensitivity and the perceived justice-orientation of collectivistic pay-for-performance practices as well as the significant positive association between overall justice sensitivity and the perceived justice-orientation of collectivistic pay-for-performance practices.

**Table 5:** Regression coefficients of justice of collectivistic pay-for-performance practices on justice sensitivities.

	Model 1				Model 2	
	*B*	*β*	*SE B*	*B*	*β*	*SE B*
Constant	2.12^***^		0.18	2.02^***^		0.23
Overall JS	0.21^***^	0.23^***^	0.05	0.21^***^	0.23^***^	0.06
Preference equity ^a^				0.04^†^	0.10^†^	0.02
Gender ^b^				−0.14^†^	−0.11^†^	0.08
Academic unit ^c^				−0.06	−0.05	0.07
*R^2^*	0.05^***^			0.06^***^		
*ΔR^2^*				0.02^†^		
Constant	2.10^***^		0.19	2.03^***^		0.23
Self-oriented JS	0.06	0.08	0.05	0.04	0.05	0.05
Other-oriented JS	0.16^**^	0.19^**^	0.05	0.17^***^	0.22^***^	0.05
Preference equity ^a^				0.04^†^	0.10^†^	0.02
Gender ^b^				−0.14^†^	−0.12^†^	0.08
Academic unit ^c^				−0.07	−0.06	0.08
*R^2^*	0.06^***^			0.08^***^		
*ΔR^2^*				0.03^†^		

^a^Preference for the equity principle. ^b^Man = 0, woman = 1. ^c^Industrial relations = 0, business school = 1. JS, justice sensitivity. ^*^p < 0.05. ^**^p < 0.01. ^***^p < 0.001.

## Discussion

The question of how human resource management practices are perceived and interpreted was addressed in this study through the lens of justice sensitivity. Overall, higher justice sensitivity was associated with perceiving human resource management practices as conveying principles of organizational justice. This is consistent with the prevailing view that human resource management practices provide clear and consistent rules and procedures for the collective. This finding also supports the idea that justice sensitivity offers a valuable perspective for the understanding of justice-based assessments of structural cues that convey the justice of an organization. In addition, this shows that not everyone is equally sensitive to the fairness aspects of the procedures and entities that they deal with (Wiesenfeld et al., 2007; Cooper and Scandura, 2015). Individuals high in justice sensitivity value justice highly and therefore see more justice in human resource management practices.

The distinction between justice for self and justice for others highlights an important individual difference that can influence perceptions of human resource management practices (Nishii et al., 2008; Wang et al., 2019). Compared to justice for self, other-oriented justice sensitivity was more strongly related to perceptions of the justice-orientation of human resource management practices overall as well as of a subset of collectivistic pay-for-performance practices.

The predictive power of justice sensitivity relative to that of socially constructed or acquired individual differences (i.e., preference for the equity principle, gender) was also demonstrated. Whereas preference for the equity principle, gender, and academic unit were not significantly associated with overall perceptions of the justice-orientation of human resource management practices, they did relate significantly to perceptions regarding the justice-orientation of pay-for-performance practices. Justice sensitivity and its perspectives (i.e., self and other) nonetheless provided additional meaningful interpretations of such perceptions. Taken together, these findings support that justice sensitivity is a valuable construct (Baumert and Schmitt, 2016) that adds a layer of understanding to current theorizing about how human resource management practices are experienced.

### Implications for research

This study has implications for both human resource management and organizational justice research. A first implication for human resource management research relates to how various policies and practices are perceived and interpreted. Our findings support research showing individual variability in how human resource management practices are perceived (Nishii et al., 2008; Wang et al., 2019). Individuals vary in the extent to which they interpret human resource management practices as more or less justice-oriented. This suggests that beyond considering ability, motivation, and opportunity, research may also consider how systems of human resource management practices may best enhance fairness perceptions by providing clear and consistent rules and procedures for the collective.

A second implication for human resource management research relates to the distinction between individualistic and collectivistic orientations and their relation to practices that bestow advantages to all or to only a select group of employees. Individuals who are more sensitive to justice for others generally perceive as more justice-oriented human resource management practices that provide consistent rules and procedures for the collective. This finding applies to the perceived justice-orientation of collectivistic pay-for-performance practices that are founded on the equity principle, but that stand to benefit the collective. Hence, by testing a stable individual orientation related to justice, we were able to detect a stable justice orientation in specific human resource management practices.

A third implication for human resource management research is the need to consider the equity principle alongside concerns for justice when variable pay practices come under scrutiny. Deutsch (1975) suggested that “allocation according to the principle of equity tends to be disruptive of social relations” (p. 146). What our findings suggest is that justice sensitivity does not necessarily lead to negative perceptions of the justice-orientation of pay-for-performance. Therefore, the benefits/effort ratio does not by itself appear to be problematic for the justice sensitive, so much as the individualistic orientation of many pay-for-performance practices. Taking into account justice sensitivities thereby complements the equity (Adams, 1965) and distributive justice (Greenberg, 1986) perspectives that permeate organizational pay-for-performance research.

A first implication of this study for organizational justice research is that it elucidates entity judgments formation (*cf.*, Hollensbe et al., 2008; Rupp and Paddock, 2010; Cooper and Scandura, 2015). Holtz and Harold (2009) added that “there is a relative deficit in our understanding of overall justice perceptions” (p. 1185) and Hollensbe et al. (2008) suggested that individuals use additional decision rules to assess the fairness of entities, compared to those used to assess the fairness of events. Current measures of the overall justice of the organization (e.g., Moorman, 1991; Moorman et al., 1998) are based upon cognitively complex generalizations across situations and people. To the extent that human resource management practices largely characterize the overall justice of the organization, then their availability may provide a clearer assessment. Human resource management practices apply across situations and people and are relatively stable over time. Therefore, rather than have individuals rate “all decisions in this company” (Beugré and Baron, 2001), it might be more suitable to have them rate the availability or effectiveness of their organization’s human resource management practices. Further confirmation of this perspective would imply that overall justice evaluations of an organization are not only based on past experiences with that organization (*cf.* Ambrose and Schminke, 2009) but also on an understanding of the practices it has adopted over time to guide its present and future conduct.

A second implication for organizational justice research lies in showing that human resource management practices are important components of systemic justice (Sheppard et al., 1992; Beugré and Baron, 2001) that can convey different degrees of justice orientation. The justice sensitive in the current study also perceived more justice in equitable distribution rules that form the basis of pay-for-performance systems. Only individualistic pay-for-performance practices were not strongly predicted by justice sensitivity, suggesting that the overall justice-orientation of human resource management practices is best captured by measures that favor the collective. From the perspective of systemic justice, the overall justice of an organization can be evaluated in terms of the overall fairness of the practices it has adopted and forged over time.

A third implication of the current study for organizational justice research is that entity justice appears to relate more to actions taken toward the collective rather than to the self. The proposition that the justice sensitive perceive more justice in more human resource management practices because they provide clear and consistent rules and procedures *for the collective* was largely supported. Conversely, individuals with a self-oriented concern for justice and a focus on their own advantages or disadvantages saw more justice in individualistic pay-for-performance practices. Hence, it would appear that perceptions of the fairness of stable structural features that promote justice (i.e., human resource management practices) are most strongly determined by other-oriented justice sensitivity, that is, by a collectivistic orientation. This differs, in terms of units of analysis, from the factors that have been found to relate to the formation of event judgments, and which largely focus on perceptions of how oneself was treated in terms of outcomes, procedures, and interpersonal relations relating to a specific time-bound event.

### Implications for practice

Disagreements regarding what is regarded as just versus unjust are not only a theoretical issue in social justice research, but also a problem of practical relevance for the management of human resources. Our study highlights for practitioners that there are perspective-related differences in interpretations of human resource management practices. Even if individuals often differ in their justice judgments, this research shows that human resource management practices are generally considered oriented toward justice by individuals most sensitive to justice. In terms of which practices might be considered the most effective at improving perceptions of justice, our study suggests that the practices investigated in the current study appear to provide clear and consistent rules and procedures for the collective. The justice sensitive and especially the justice sensitive with a genuine concern for the justice of others perceive more justice in these human resource management practices than do individuals with lower or self-oriented justice sensitivity.

The practices investigated in this study were high-performance work practices (Posthuma et al., 2013) that have the potential to improve organizational performance (Combs et al., 2006). Our findings suggest that they may also contribute to justice perceptions that have a number of performance implications of their own (Cohen-Charash and Spector, 2001). To the extent that they set the stage for justice to be enacted, implementing more of these high-performance work practices may contribute to organizational performance by improving overall justice perceptions, as well as ability, motivation, and opportunity.

### Limitations

Our data was collected from university students who might lack an accurate, practical understanding of business practices. We are confident, however, that human resource management practices have some motivational potential in programs with a strong curricular emphasis in this area. In addition, almost all respondents (96%) had some work experience, with an average of 5.10 years (*SD* = 4.34), and 66% of respondents indicating that they were employed at the time of the survey. Therefore, almost all respondents would have had direct experience with human resource management practices (e.g., hiring, pay, benefits). To explore whether a sample of more experienced respondents might have rated the human resource management practices differently, we examined the relation between participant work experience and ratings of the 61 human resource management practices. Only ratings of two of the practices were significantly influenced by work experience. With a confidence interval of 95 percent (*p* < 0.05), we would expect up to three significant correlations to occur by chance so that these two significant correlations were meaningful. It thus appears that ratings of the 61 human resource management practices were not influenced by work experience.

A second limitation is the possibility of common method variance given that all variables were measured from a single source. However, concerns about common method variance were lessened by collecting data in two waves and thereby temporally separating measurement of the independent and dependent variables (Lindell and Brandt, 2000).

## Conclusion

The fairness of human resource management practices was examined in this study to better understand how individual differences in justice sensitivity influence assessments of the justice-orientation of human resource management practices. The distinction between self-oriented and other-oriented justice sensitivities was meaningful in determining reactions to human resource management practices, especially assessments of pay-for-performance practices. The findings inform current research about the meanings borne by human resource management practices and increase our understanding of the process of entity justice judgments formation. This research thereby contributes to a better understanding of how systemic justice – justice at the organizational level – is shaped by the human resource practices that guide conduct and serve as important sources of justice (Cropanzano et al., 2001).

## Data availability statement

The datasets presented in this article are not readily available because none. Requests to access the datasets should be directed to victor.haines@umontreal.ca.

## Ethics statement

The studies involving humans were approved by CERSES 20-168-D-University of Montreal. The studies were conducted in accordance with the local legislation and institutional requirements. The participants provided their written informed consent to participate in this study.

## Author contributions

VH: Conceptualization, Formal analysis, Funding acquisition, Methodology, Supervision, Writing – original draft, Writing – review & editing. DP: Conceptualization, Validation, Writing – review & editing. SG: Funding acquisition, Validation, Writing – review & editing.

## Footnotes

^1^This altruism may be socially motivated by fear of criticism (Bondü and Inerle, 2020).

^2^A search with the keywords “procedural justice” on the website of the Society for Human Resource Management (https://www.shrm.org) generated 859 results. The contents addressed employment discrimination, progressive discipline, procedural irregularities during a hiring interview and other such justice-related topics.

^3^Leventhal (1980) alternatively described the principle of equity as the merit principle or contributions rule.

^4^Although this list covers a very broad spectrum of human resource management practices, it does not include every possible human resource management practice. For instance, the practice of paying employees at least minimum wage is not included in this set of practices.
